# Cost-effectiveness of an electronic clinical decision support system for improving quality of antenatal and childbirth care in rural Tanzania: an intervention study

**DOI:** 10.1186/s12913-017-2457-z

**Published:** 2017-08-07

**Authors:** Happiness Pius Saronga, Els Duysburgh, Siriel Massawe, Maxwell Ayindenaba Dalaba, Peter Wangwe, Felix Sukums, Melkizedeck Leshabari, Antje Blank, Rainer Sauerborn, Svetla Loukanova

**Affiliations:** 1 Muhimbili University of Health and Allied Sciences, Dar es Salaam, Tanzania; 2 Navrongo Health Research Centre, Navrongo, Ghana; 3 Department of Clinical Pharmacology and Pharmacoepidemiology, University of Heidelberg, Heidelberg, Germany; 4 Institute of Public Health, University of Heidelberg, Heidelberg, Germany; 5 Department of General Medicine and Implementation Research, University of Heidelberg, Heidelberg, Germany; 6 International Centre for Reproductive Health (ICRH), Ghent University, Ghent, Belgium

**Keywords:** Clinical decision support system, Pharmacoeconomics, Cost-effectiveness analysis, Antenatal care, Childbirth care, Rural health care, Medical health information technology, Tanzania

## Abstract

**Background:**

QUALMAT project aimed at improving quality of maternal and newborn care in selected health care facilities in three African countries. An electronic clinical decision support system was implemented to support providers comply with established standards in antenatal and childbirth care. Given that health care resources are limited and interventions differ in their potential impact on health and costs (efficiency), this study aimed at assessing cost-effectiveness of the system in Tanzania.

**Methods:**

This was a quantitative pre- and post- intervention study involving 6 health centres in rural Tanzania. Cost information was collected from health provider’s perspective. Outcome information was collected through observation of the process of maternal care. Incremental cost-effectiveness ratios for antenatal and childbirth care were calculated with testing of four models where the system was compared to the conventional paper-based approach to care. One-way sensitivity analysis was conducted to determine whether changes in process quality score and cost would impact on cost-effectiveness ratios.

**Results:**

Economic cost of implementation was 167,318 USD, equivalent to 27,886 USD per health center and 43 USD per contact. The system improved antenatal process quality by 4.5% and childbirth care process quality by 23.3% however these improvements were not statistically significant. Base-case incremental cost-effectiveness ratios of the system were 2469 USD and 338 USD per 1% change in process quality for antenatal and childbirth care respectively. Cost-effectiveness of the system was sensitive to assumptions made on costs and outcomes.

**Conclusions:**

Although the system managed to marginally improve individual process quality variables, it did not have significant improvement effect on the overall process quality of care in the short-term. A longer duration of usage of the electronic clinical decision support system and retention of staff are critical to the efficiency of the system and can reduce the invested resources. Realization of gains from the system requires effective implementation and an enabling healthcare system.

**Trial registration:**

Registered clinical trial at www.clinicaltrials.gov (NCT01409824). Registered May 2009.

**Electronic supplementary material:**

The online version of this article (doi:10.1186/s12913-017-2457-z) contains supplementary material, which is available to authorized users.

## Background

Poor quality of health care is among the causes of high maternal and newborn morbidity and mortality burden in Tanzania. Although the maternal mortality ratio in Tanzania has been slowly declining over the past years, it is still estimated to be at a staggering 398 deaths per 100,000 live births [1]. Neonatal mortality rate is 21 per 1000 live births and the lifetime risk of maternal death is 1 in 45 [1, 2].

One potential reason for poor quality of health care is the existence of a “know-do gap”, whereby health workers do not perform to the best of their knowledge [3, 4]. Poor performance is very often a result of low motivation among the health care providers compounded by shortage of financial and well trained human resources, low retention of staff, shortage of infrastructure and supplies, poor transport and communication infrastructure, weak governance and management [5, 6]. This is a sensitive problem in the maternal and newborn health care provision. The lack of motivation leads to an insufficient translation of knowledge into optimal utilization of resources in the health sector (know-do gap) and to the provision of suboptimal maternal and newborn health care.

The “know-do gap” represents a challenge that must be addressed to strengthen quality in the maternal and newborn care in an effort to reduce the maternal and newborn morbidity and mortality in Tanzania. QUALMAT (quality of maternal care) was a project, which aimed at improving the quality of maternal and newborn care (MNC) by addressing the “know-do gap” among health care workers in selected health facilities in Lindi Rural District, Tanzania. It specifically aimed at increasing provider performance by developing and implementing an electronic clinical decision support system (eCDSS) in order to help providers comply with established standards of care in antenatal and childbirth care. A previous study had found poor quality of care among health care providers in these health facilities [7].

An eCDSS provides patient-specific recommendations to support decision making. eCDSSs have been shown to be effective and sustainable for maintaining good clinical practice [8–14]. Different tools have been tested and showed positive results in improving quality of care provided to clients [12], reducing medical errors and adverse events [15, 16], improving prescriptions [15], increasing adherence to guidelines [17], aiding preventive care [8, 17, 18] and reducing cost [14, 17]. The eCDSS in QUALMAT project was developed to improve performance of health workers by facilitating and enforcing adherence to World Health Organization (WHO) guidelines on maternal care and adapted to local standards [7]. Increased guidelines adherence – such as early prevention, risk detection, proper treatment and referral - should ultimately improve quality of maternal health care services [11].

Because of competing demands for limited health care resources and due to a different potential to impact on health and costs (efficiency), economic evaluation of new intervention, such as the eCDSS is indispensable. This will allow to choose the best and most cost-effective options for implementation [19]. Most cost-effectiveness studies related to medical health information technologies have been conducted in developed countries where they have been used more often. A number of these assessments have shown such systems are both medically and cost effective [20–24]. Medical health information technology is increasingly used in many developing countries but there is scarcity of cost-effectiveness studies in literature covering this context. Therefore this study aimed at assessing cost-effectiveness of the eCDSS compared to the standard paper-based maternal and neonatal care services, which were in use at the study sites.

## Methods

### Study area

This study is a sub-study to the European Union funded research project QUALMAT. The QUALMAT study was conducted in 3 African countries - Burkina Faso, Ghana and Tanzania. This sub-study was carried out in the Tanzanian sites, six health facilities in Lindi rural district, where the eCDSS was implemented.

Lindi District Council is within Lindi region in southern Tanzania covering 7538 km^2^ of land. It has 194,143 people- 91,647 males and 102,496 females [25]. Lindi region is predominantly rural with a principally agrarian economy. Although the region has good economic potential in terms of natural resources, its population has incomes around the poverty line and a large burden of maternal and neonatal morbidity and mortality [26–28]. Household average size is around 3.7 members, with subsistence agriculture as the main economic activity. The district has 1 hospital, 6 health centers and 38 dispensaries in total. Dispensaries and health centers offer the most basic health services including MNC (antenatal care and childbirth up to 24 h stay). Complicated cases are referred to district or regional hospitals.

Most women (99.6%) in this district get at least one skilled antenatal care assessment. Fifty percent of births are assisted by skilled birth attendant, a little over 50% of births occur in health facilities and a little over 40% receive postnatal care within 2 days of childbirth [29]. Infant and neonatal mortality rates were estimated at 76.4 and 43.2 per 1000 live births respectively [30]. According to the Ministry of Health, maternal mortality ratio and infant mortality rate were 141 per 100,000 live births and 71.5 per 1000 live births respectively in 2011 [28].

### Study design

This was a quantitative pre- and post- intervention study. The intervention district was Lindi Rural where a computer-assisted eCDSS was implemented in 6 health centers between April 2012 and April 2014 for management of maternal health care (antenatal care (ANC) and childbirth). Lindi district was selected for this study because of the availability of emergency obstetric and newborn services in the district.

One private and five public health centers were chosen from the district based on inclusion criteria. First, they were representative for the situation of rural primary health care sites in a particular disadvantaged area of Tanzania. Second, they met national standards for medical infrastructure, equipment and staffing and third, routine antenatal and childbirth care services were available [31].

### The QUALMAT eCDSS

The eCDSS was stand-alone, java-based software based on the Integrated Management of Pregnancy and Childbirth Care guideline of the WHO adapted to local standards [32]. It provided computerized guidance and clinical decision support for routine antenatal and childbirth care up to 24 h post-childbirth. It provided guidance through routine actions in maternal and perinatal care- supported history taking, physical examination, basic laboratory tests, as well as provision of counseling and preventive measures; it integrated clinical data to detect situations of concern by algorithms. Entered data were screened and diagnoses or alerts were issued to inform the user about possible medical situations of concern that required consideration during the visit. It provided electronic tracking of peri- and post-natal activities, using an electronic Partograph with continuous monitoring of the delivery process on the screen. Additional details on eCDSS conception, development, adoption, usability, as well as integration into clinical workflow has been published elsewhere [11, 33–35].

### Data collection and analysis

#### Cost of antenatal and childbirth care before and after eCDSS implementation

The cost study was cross-sectional quantitative and was conducted retrospectively. Both pre-intervention and post-intervention data were collected and analyzed from a health care provider’s perspective. Cost data of health service provision were collected using structured questionnaire through document reviews, interviews and physical inventory of resources used at the facilities. Pre-intervention cost data was collected between November and December 2010, the collected data was for the year 2009 representing cost of resources used to provide health services for the whole year. Post-intervention cost data was collected between April and May 2014 representing cost of resources used to provide health services for the year 2013.

Wherever possible, unit prices of resources were collected directly from health facilities, for example drugs, medical equipment and supplies, personnel (including salaries, benefits, allowances and training) and transport. Some cost data was gathered from the Medical Stores Department (MSD), Expanded Program on Immunization (EPI) and District Medical Officer’s (DMO) offices. In some cases prices were imputed from market sources, for example prices of some locally obtained equipment including benches, chairs, television set, buckets etc. Building costs were based on estimated replacement cost in these rural areas [36]. Data were entered, cleaned and analysed using Microsoft Office Excel.

Step-down cost accounting technique was used to estimate costs. Using this technique a range of resources needed to run a facility were identified and then assigned to chosen cost centers on an allocation basis. The costs in each cost center were aggregated together in overarching themes. This methodology was adopted from Conteh and Walker [37] and has been used in various studies [38–42]. The average percentage of time spent on activities by staff was the cost allocation basis.

All costs were identified in local currency, Tanzanian Shillings (TZS), and later converted to United States Dollars (USD) according to the average exchange rate. 1 USD = 1326 TZS for pre-intervention cost analysis (2009) and 1 USD = 1676 TZS for post-intervention cost analysis (2013).

Annuitization was applied to estimate the equivalent annual costs (EAC) for capital outlays. We used the following annualization formula by Drummond et al. [43].

$$ K=\frac{E}{\left[1+r\right]}+\frac{E}{{\left[1+r\right]}^2}+\cdots \cdots +\frac{E}{{\left[1+r\right]}^n} $$

Where; *K* is capital outlay*, E* is equivalent annual cost*, r* is discount rate and *n* is number of useful years for the capital item. 3% discount rate and useful life of 10 years were used for equipment and vehicle cost annualization. For buildings the same discount rate was used but a useful life of 30 years was considered more appropriate for our context. The discount rate was chosen per international economic evaluation guidelines. Useful years were chosen as applied in similar studies by Mills et al. [44].

More details on MNC cost data collection and analysis can be found in a previously published paper [36].

For comparability between the pre- and post-intervention cost estimates, we adjusted the pre-intervention cost estimates for inflation using the consumer price index (CPI). CPI for Tanzania in 2009 was 94.2 and in 2013 was 141.0 [45]. We used the following adjustment formula [46]:

$$ {Y}_B={Y}_P\left[\frac{D_B}{D_P}\right] $$

Where:

*Υ*_*B*_= base year value

*Υ*_*P*_= past year value

*D*_*B*_= index value of base year

*D*_*P*_= index value of past year

### Cost of installing and operating the eCDSS

Data for cost of installing and operating the eCDSS was collected retrospectively from the program (QUALMAT) perspective. This data represent the cost of all the resources (purchased and donated) used during eCDSS installation and operation 2012-14. Data were retrieved from project accounts records using a cost collection sheet and interviews with project personnel. In cases where resources were donated to QUALMAT (not purchased using QUALMAT funds thus not indicated in the program accounts records) we adopted market prices for similar resources. eCDSS intervention costs were estimated using Ingredients approach. Broadly the cost consisted of the following items; eCDSS software, vehicle, computers, furniture, facility (space and electricity), transport, personnel (salaries and allowances), short-term computer and eCDSS training, supplies and communication.

We estimated financial costs and economic costs separately. Financial costs are the real-money outlays for resources required to produce an intervention and to manage patient’s health outcome, while economic costs of an intervention are the opportunity costs of the resources used to implement the intervention [46]. In financial cost analysis therefore the costs reflected how much and when the money was spent during eCDSS intervention by QUALMAT, while in economic cost analysis the costs in addition reflected the cost of donated goods and services (health workers time and facility cost) used and the equivalent annual cost of capital items.

The economic costs were further categorized into fixed costs (costs that do not change with output level) and variable costs (costs that change proportionately with output level). eCDSS cost per MNC contact was calculated by dividing total economic cost of eCDSS intervention by the total number of MNC contacts (ANC visits plus number of childbirths) using eCDSS.

Costs in local currency (Tanzanian Shillings- TZS) were converted into United States Dollars (USD). The exchange rates ranged between 1 USD = 1410 TZS to 1 USD = 1676 TZS in the period 2010 and 2013 [47]. Excel spreadsheet was used for data analysis.

More details on eCDSS cost data collection and analysis can be found in a previously published paper [48].

### Effectiveness of the eCDSS

Process quality of maternal care is the effectiveness (outcome) measure in our analysis. Success of the eCDSS intervention was measured through quality of care assessment, which compared quality of maternal care before and after intervention [7, 49]. To assess process quality, structured questionnaires with checklists were used to collect information through observations. Variables were grouped into topic groups based on the WHO guidelines and taking into consideration different dimensions of care, interpersonal, technical performance and continuity of care. A variable would receive a score of 1 if the activity was observed and performed according to accepted standards of care and a score of 0 if the activity was not observed or not performed according to accepted standards of care.

The study population comprised women undergoing antenatal consultation, women undergoing childbirth, newborns (in the first hours/days of stay at a health facility after childbirth) and health workers providing ANC and childbirth services. The sample size was calculated assuming independence between the observations, 80% statistical power, significance level of 0.05, standard deviation of 0.4 and normal distribution of quality scores; roughly 200 ANC observations and 200 childbirth observations.

Pre-intervention data collection was done between June and November 2010 and post-intervention data collection was done between October 2013 and April 2014. This was done by trained certified nurses and midwives who had no links to the health facilities. Data collectors were interchanged from time to time to ensure objective observations.

Epi Info version 3.5.1 was used for data entry and Stata/IC 11.2 was used for data analysis. Quality scores were calculated for ANC and childbirth. Arithmetic mean for topic groups was calculated and used as a quality of care score. The Wilcoxon-Mann-Whitney test (rank-sum test) was used to compare quality scores before and after intervention. Results were assessed for statistical significant differences using *p* < 0.05. Additional details about quality assessment is described elsewhere [7, 49].

We calculated percentage change in process quality score before and after eCDSS implementation. We used the percentage change formula:

$$ \% changeprocessquality=\frac{Q_2-{Q}_1}{Q_1}\times 100 $$

Where; *Q*_1_ is process quality of MNC before eCDSS implementation.

*Q*_2_ is process quality of MNC after eCDSS implementation.

### Cost-effectiveness of the eCDSS

We calculated incremental cost-effectiveness ratio (ICER), which represents the additional cost of one unit of outcome gained by one strategy compared with another [43, 46]. The ICER is expressed as the ratio of the difference in cost to the difference in effectiveness between the two comparators. Here we compare the eCDSS and the standard paper-based approach to MNC at the primary health care facilities. We used the following formula to calculate ICER:

$$ ICER=\frac{\varDelta C}{\varDelta E}=\frac{C_B-{C}_A}{E_B-{E}_A} $$

Where; *C*_*A*_ is the cost of intervention A (intervention currently in place)

*E*_*A*_ is the effectiveness of intervention A (intervention currently in place)

*C*_*B*_ is the cost of intervention B (new intervention under consideration for adoption)

*E*_*B*_ is the effectiveness of intervention B (new intervention under consideration for adoption)

The eCDSS is the new intervention B, while the existing paper-based system for ANC and childbirth is intervention A. The current intervention is also known as “do-nothing” as it involves only the conventional routine activities.

The ICER analysis used pre- and post-intervention cost and process quality data and eCDSS cost data. These are already described in previous sections. Therefore:

*C*_*B*_= post-intervention MNC average cost plus average cost of eCDSS implementation

*E*_*B*_= post-intervention mean process quality score

*C*_*A*_= pre-intervention MNC average cost

*E*_*A*_= pre-intervention mean process quality score

We calculated ICERs for ANC and childbirth separately and in each we tested four models. In model 1 we used pre-intervention cost unadjusted for inflation and financial eCDSS cost. In model 2 we used pre-intervention cost adjusted for inflation and financial eCDSS cost. In model 3 we used pre-intervention cost unadjusted for inflation and economic eCDSS cost and in model 4 we used pre-intervention cost adjusted for inflation and economic eCDSS cost. The aim was to illustrate the differences brought about by adjustment of costs (inflation adjustment, annualization and shadow pricing) on final estimates.

One-way sensitivity analysis was conducted to determine whether changes in process quality score and cost would impact the ICERs. These were altered by a 10% increase or decrease.

We compared the health facility ICERs to WHO cost-effectiveness thresholds. According to the threshold, a ratio is very cost-effective if is less than the country’s per capita gross domestic product (GDP), cost-effective if it is one to three times greater than the country’s per capita GDP and not cost-effective if it is more than three times greater than the country’s per capita GDP [50]. Per capita GDP based on current prices (USD) for Tanzania was between 998.1 and 1005.6 in 2014 [51, 52]. While per capital GDP based on purchasing power parity (current international dollar) was between 2591.2 and 2666.7 [52, 53]. We also compared the results to other interventions used to improve quality of maternal health care in developing countries.

### Ethics statement

QUALMAT research project was granted ethical approval from the Muhimbili University of Health and Allied Sciences, Tanzania, ethics committee (reference number MU/RP/AEC/Vol.XIII/1) and the Faculty of Medicine of the University of Heidelberg, Germany, ethics commission (reference number S173/2008). Likewise, informed consent (written or verbal) was sought from all participants.

## Results

### Health facility MNC cost

Total facility cost in the intervention sites before eCDSS implementation totalled 352,749 USD with an average total cost per health centre at 58,792 USD (Table 1). In 2009 ANC consumed an average of 7140 USD per health centre and childbirth care consumed 7389 USD per health centre. Post-intervention total facility cost amounted to 560,556 USD with an average cost per health centre at 93,426 USD, ANC cost per health centre was 6811 USD and childbirth care cost per health centre was 6107 USD.

**Table 1:** Health facility cost distribution pre-post intervention (in USD)

	Pre-intervention (2009)				Post-intervention (2013)			
Health Facility	ANC	Childbirth	Other	Total	ANC	Childbirth	Other	Total
HC1	9076.39	5498.20	53,899.93	68,474.52	7889.38	7236.92	95,244.18	110,370.48
HC2	11,308.74	7747.34	43,585.53	62,641.61	4979.58	3853.87	88,192.45	97,025.90
HC3	8105.92	8800.22	48,038.27	64,944.41	7958.49	8432.57	84,163.51	100,554.57
HC4	8624.94	11,845.95	35,341.16	55,812.04	5605.24	5409.58	75,549.87	86,564.69
HC5	1501.62	6981.17	35,177.52	43,660.32	3656.48	2990.99	53,176.31	59,823.78
HC6	4223.83	3464.21	49,528.56	57,216.61	10,776.72	8719.36	86,720.54	106,216.62
Total Cost	42,841.44	44,337.10	265,570.97	352,749.51	40,865.89	36,643.28	483,046.87	560,556.04
Average Cost (inflation adjusted to 2013)	7140.24 (10,687.62)	7389.52 (11,060.74)	44,261.83 (66,251.78)	58,791.59 (88,000.14)	6810.98	6107.21	80,507.81	93,426.01

*HC* Health Centre

### Activity time allocation in the post-intervention study

On average, about 7% (range 5-9%) and 6% (range 4-7%) of staff time was spent to provision of ANC and childbirth services respectively. The rest of time was divided between other activities (Table 2). This represents an average of 54% and 46% of MNC time for ANC and childbirth respectively.

**Table 2:** Post-intervention personnel reported time allocation by health facility and activity in percentage, 2013

Health Facility	Administration	Transport	Pharmacy and lab	ANC	Childbirth	Other
HC1	4.4	8.3	7.7	8.8	6.7	64.1
HC2	3.3	0.0	4.0	5.3	4.1	83.3
HC3	4.2	9.1	3.3	6.4	5.7	71.4
HC4	5.1	7.7	5.2	6.8	7.2	68.0
HC5	6.6	15.4	7.3	5.1	3.6	62.0
HC6	6.0	5.9	6.4	6.4	5.6	69.6
Average	4.9	7.7	5.6	6.5	5.5	69.7

*HC* Health Center

### Cost of eCDSS installation and operation

Total financial cost of eCDSS intervention in all the six health centers amounted to 209,085 USD, equivalent to 34,848 USD per health center (see Additional file 1). In estimating economic cost, we went a step further and included estimates on the donated health workers’ time and facility (space and electricity), and also annualized capital costs as explained in the methods section. Total economic cost of eCDSS intervention in all the six health centers amounted to 167,318 USD, equivalent to 27,886 USD per health center, a difference of 19.9% from the total financial cost above (see Additional file 2). Most costs were incurred during start-up with large investment in capital inputs and training. Training and personnel made the largest cost components apart from the eCDSS software. More than three-quarters of the total economic cost were fixed- 90.9%, while variable costs were only 9.1% (Table 3). Total MNC contacts registered in the eCDSS were 3888 (74% of all MNC contacts) therefore economic cost per eCDSS contact was 43.03 USD. Some of these results have been published [48].

**Table 3:** Fixed, variable and average costs of eCDSS intervention in USD, 2009-14

Item	Total	Percentage
Fixed Cost (eCDSS)	152,214.01	90.9
Variable Cost (eCDSS)	15,104.17	9.1
Total Economic Cost (eCDSS)	167,318.18	100.0
Total ANC contacts registered at study sites (2 years)	3802	100.0
Total ANC contacts using eCDSS (2 years)	2703	71.0
Total childbirths registered at study sites (2 years)	1427	100.0
Total childbirths using eCDSS (2 years)	1185	83.0
Total eCDSS contacts (ANC plus childbirths using eCDSS)	3888	
Cost per eCDSS contact (Total Economic Cost (eCDSS)/Total eCDSS contacts)	43.03	

### Effectiveness of the eCDSS

#### Quality of ANC before and after eCDSS implementation

Table 4 shows ANC process quality scores before and after eCDSS implementation [7, 54]. The overall quality went up from 0.88 to 0.92 representing a 4.5% change however this change was not statistically significant (*p*-value = 0.75). Considering specific topic groups, it is clear that technical performance overall has improved from 0.71 to 0.77 however this was not statistically significant (*p*-value = 0.92). Almost all variables under this group did improve but none was significant. The quality of laboratory examination went down post-intervention by 0.10 however this fall was not statistically significant (*p*-value = 0.25).

**Table 4:** Quality of ANC pre- and post-intervention (*n* = 6 health centers)

Quality Indicator	Pre-intervention	Post-intervention	*p*-value	Difference	% change
1. Technical performance	0.71	0.77	0.92		
history taking	0.71	0.87	0.05		
clinical examination	0.86	0.92	0.35		
laboratory examination	0.54	0.44	0.25		
preventive measures	0.86	0.89	0.17		
counselling	0.54	0.68	0.60		
management and treatment	0.77	0.83	0.92		
2. Inter-personal performance	0.96	1	0.09		
3. Continuity of care	0.98	1	0.04		
Total ANC observation quality score	0.88	0.92	0.75	0.04	4.53

Inter-personal performance also improved from 0.96 to 1 but the difference was not statistically significant (*p*-value = 0.09). Continuity of care had a significant positive difference between pre and post- intervention with the score rising from 0.98 to 1 (*p*-value = 0.04).

#### Quality of childbirth care before and after eCDSS implementation

Overall, childbirth care process quality improved post-intervention from 0.64 to 0.79, representing a 23% change, however this was not statistically significant (*p*-value = 0.07) (Table 5) [7, 54]. Again looking at specific topic groups, in general there was an improvement in technical performance by 0.09 but not statistically significant (*p*-value = 0.46). Just like with ANC, almost all variables under this group had an improvement in quality, however none had a statistically significant improvement. Newborn monitoring fell in quality by 0.15 but this was not statistically significant (*p*-value = 0.92).

**Table 5:** Quality of childbirth pre and post-intervention (*n* = 6 health centers)

Quality Indicator	Pre-intervention	Post-intervention	*p*-value	Difference	% change
1. Technical performance	0.65	0.74	0.46		
history taking	0.72	0.87	0.12		
clinical examination on admission	0.66	0.71	0.25		
monitoring mother	0.3	0.59	0.05		
monitoring new-born	0.62	0.47	0.92		
care and examination mother	0.5	0.58	0.60		
care and examination new-born	0.73	0.83	0.25		
delivery new-born	0.87	0.91	0.92		
delivery placenta	0.81	0.9	0.92		
counselling	0.65	0.83	0.25		
2. Inter-personal performance	0.79	0.88	0.05		
3. Recording	0.49	0.76	0.05		
Total childbirth observation quality score	0.64	0.79	0.07	0.15	23.32

Inter-personal performance improved by 0.09 however not statistically significant (*p*-value = 0.05). Recording also improved by 0.27 however also not statistically significant (*p*-value = 0.05).

### Cost-effectiveness of the eCDSS

#### Cost-effectiveness of eCDSS in ANC

Using reported staff allocation time, the average time spent in ANC post-intervention was 54% of the total time health workers spent in providing maternal and newborn care. Therefore 54% of the average cost of eCDSS implementation (34,848USD financial costs and 27,886 USD economic costs) was used in the calculation of ICER for ANC. While the average time spent on childbirth was 46%. Therefore 46% of the average cost of eCDSS implementation was used in the calculation of ICER for childbirth.

In model 1, pre-intervention cost for ANC was 7140 USD (cost unadjusted for inflation) and post-intervention cost for ANC was 25,629 USD (post-intervention facility cost plus eCDSS financial cost). Quality of ANC increased by 4.5% between pre and post-intervention giving an incremental cost effectiveness ratio (ICER) of 4083 USD, representing dollar cost of 1% change in ANC process quality per health center (Table 6).

**Table 6:** Incremental cost-effectiveness analysis of eCDSS in ANC

	Average ANC cost (USD)	Incremental cost (USD)	Quality of ANC	Incremental quality (%)	Incremental cost effectiveness ratio (USD)
Model 1: Pre-intervention cost unadjusted for inflation, financial eCDSS cost					
Pre	7140.24		0.88		
Post	25,628.64	18,488.40	0.92	4.53	4083
(including average eCDSS cost)	(6810.98 + 18,817.66)				
Model 2: Pre-intervention cost adjusted for inflation, financial eCDSS cost					
Pre	10,687.62		0.88		
Post	25,628.64	14,941.02	0.92	4.53	3299
(including average eCDSS cost)	(6810.98 + 18,817.66)				
Model 3: Pre-intervention cost unadjusted for inflation, economic eCDSS cost					
Pre	7140.24		0.88		
Post	21,869.62	14,729.38	0.92	4.53	3253
(including average eCDSS cost)	(6810.98 + 15,058.64)				
Model 4: Pre-intervention cost adjusted for inflation, economic eCDSS cost					
Pre	10,687.62		0.88		
Post	21,869.62	11,182.00	0.92	4.53	2469
(including average eCDSS cost)	(6810.98 + 15,058.64)				

In model 2, pre-intervention cost for ANC was 10,688 USD (cost adjusted for inflation) and post-intervention cost for ANC was 25,629 USD (post-intervention facility cost plus eCDSS financial cost). Quality of ANC increased by 4.5% between pre and post-intervention giving an incremental cost effectiveness ratio (ICER) of 3299 USD, representing dollar cost of 1% change in ANC process quality per health center.

In model 3, pre-intervention cost for ANC was 7140 USD (cost unadjusted for inflation) and post-intervention cost for ANC was 21,869 USD (post-intervention facility cost plus eCDSS economic cost). Quality of ANC increased by 4.5% between pre and post-intervention giving an incremental cost effectiveness ratio (ICER) of 3253 USD, representing dollar cost of 1% change in ANC process quality per health center.

In model 4, pre-intervention cost for ANC was 10,688 USD (cost adjusted for inflation) and post-intervention cost for ANC was 21,869 USD (post-intervention facility cost plus eCDSS economic cost). Quality of ANC increased by 4.5% between pre and post-intervention giving an incremental cost effectiveness ratio (ICER) of 2469 USD, representing dollar cost of 1% change in ANC process quality per health center.

#### Sensitivity analysis

Sensitivity analysis shows that the ICER would change when quality scores and cost change. Results show that it would range between 2222 USD and 4535 USD (Table 7).

**Table 7:** Sensitivity analysis results for ANC ICERs (in USD)

	ICER (quality score change)	ICER (post-intervention cost change)
Model 1		
10% increase	3710.30	4489.46
10% decrease	4534.81	3673.19
Model 2		
10% increase	2998.40	3628.06
10% decrease	3664.71	2968.41
Model 3		
10% increase	2955.93	3576.67
10% decrease	3612.80	2926.37
Model 4		
10% increase	2244.03	2715.28
10% decrease	2742.70	2221.59

#### Cost-effectiveness of eCDSS in childbirth

In model 1, pre-intervention cost for childbirth was 7389 USD (cost unadjusted for inflation) and post-intervention cost for childbirth was 22,137 USD (post-intervention facility cost plus eCDSS financial cost). Quality of childbirth increased by 23% between pre and post-intervention giving an incremental cost-effectiveness ratio (ICER) of 633USD, representing dollar cost of 1% change in childbirth process quality per health center (Table 8).

**Table 8:** Incremental cost-effectiveness analysis of eCDSS in childbirth

	Average Childbirth cost (USD)	Incremental cost (USD)	Quality of Childbirth care	Incremental quality (%)	Incremental cost effectiveness ratio (USD)
Model 1: Pre-intervention cost unadjusted for inflation, financial eCDSS cost					
Pre	7389.52		0.64		
Post	22,137.07	14,747.55	0.79	23.32	633
(including average eCDSS cost)	(6107.21 + 16,029.86)				
Model 2: Pre-intervention cost adjusted for inflation, financial eCDSS cost					
Pre	11,060.74		0.64		
Post	22,137.07	11,076.33	0.79	23.32	475
(including average eCDSS cost)	(6107.21 + 16,029.86)				
Model 3: Pre-intervention cost unadjusted for inflation, economic eCDSS cost					
Pre	7389.52		0.64		
Post	18,934.94	11,545.42	0.79	23.32	495
(including average eCDSS cost)	(6107.21 + 12,827.73)				
Model 4: Pre-intervention cost adjusted for inflation, economic eCDSS cost					
Pre	11,060.74		0.64		
Post	18,934.94	7874.20	0.79	23.32	338
(including average eCDSS cost)	(6107.21 + 12,827.73)				

In model 2, pre-intervention cost for childbirth was 11,061 USD (cost adjusted for inflation) and post-intervention cost for childbirth was 22,137 USD (post-intervention facility cost plus eCDSS financial cost). Quality of childbirth increased by 23% between pre and post-intervention giving an incremental cost effectiveness ratio (ICER) of 475 USD, representing dollar cost of 1% change in childbirth process quality per health center.

In model 3, pre-intervention cost for childbirth was 7389 USD (cost unadjusted for inflation) and post-intervention cost for childbirth was 18,935 USD (post-intervention facility cost plus eCDSS economic cost). Quality of childbirth increased by 23% between pre and post-intervention giving an incremental cost effectiveness ratio (ICER) of 495 USD, representing dollar cost of 1% change in childbirth process quality per health center.

In model 4, pre-intervention cost for childbirth was 11,061 USD (cost adjusted for inflation) and post-intervention cost for childbirth was 18,935 USD (post-intervention facility cost plus eCDSS economic cost). Quality of childbirth increased by 23% between pre and post-intervention giving an incremental cost effectiveness ratio (ICER) of 338 USD, representing dollar cost of 1% change in childbirth process quality per health center.

#### Sensitivity analysis

Sensitivity analysis results show that the ICER would change when quality scores and cost change. Results show that it would range between 304 USD and 703 USD (Table 9).

**Table 9:** Sensitivity analysis results for childbirth ICERs, in USD

	ICER (quality score change)	ICER (post-intervention cost change)
Model 1		
10% increase	575.01	695.76
10% decrease	702.78	569.26
Model 2		
10% increase	431.86	522.56
10% decrease	527.83	427.55
Model 3		
10% increase	450.15	544.69
10% decrease	550.19	445.65
Model 4		
10% increase	307.01	371.49
10% decrease	375.24	303.94

## Discussion

This study investigated cost-effectiveness of an electronic clinical decision support system in improving process quality of ANC and childbirth care in 6 rural health centers of Tanzania. Findings show that total financial cost of eCDSS installation and operation for 24 months in a health centre was 34,848 USD, while economic cost was 27,886 USD. Clinical effectiveness evaluation of the eCDSS shows that process quality of ANC and childbirth care improved by 4.5% and 23.2% respectively post-intervention; however these improvements were not statistically significant. Incremental cost-effectiveness ratios of the eCDSS depend on assumptions made on cost and quality. ANC base-case ICER ranges between 2469 USD to 4083 USD per 1% change in ANC process quality. Base-case childbirth ICER ranges between 338 USD and 633 USD per 1% change in childbirth process quality.

Even though the eCDSS did not manage to significantly improve the overall process quality of ANC and childbirth care, there were marginal positive changes in ANC history taking (marginally significant at *p* < 0.1), interpersonal performance (marginally significant at *p* < 0.1) and continuity of care (significant at *p* < 0.05). Maternal monitoring, interpersonal performance and recording during childbirth also marginally improved (at *p* < 0.1).

Proper history taking ensures that women are correctly attended by health workers during pregnancy and also avoids repetitive history taking by subsequent health care providers. It can aid timely identification of risky pregnancies and facilitate timely intervention. Moreover, history taking provides a learning opportunity for health workers and their clients facilitating informed choice. History taking can also strengthen interpersonal communication, continuity of care and patient/client records. Continuity of care entails a combination of services given in ANC. The eCDSS can improve the continuum of care facilitating timely identification of risky pregnancies and offer timely intervention including referrals therefore improving maternal and neonatal outcomes [55].

Maternal and newborn monitoring is generally of poor quality at the study sites [7, 54]. Critical shortage of skilled human resources, shortage of evidence-based MNC guidelines and low or incomplete usage of Partograph during childbirth can limit proper monitoring [56]. The eCDSS can help improve foetal, maternal and newborn monitoring of danger signs during labour and delivery, for example by integrating information from inbuilt Partograph and maternal history in decision making. Proper monitoring can improve maternal and neonatal outcomes by facilitating early intervention in case of complications.

Through eCDSS on-screen prompts a health worker is nudged to maintain good communication with clients reflecting patient-centeredness of care. Communication is a very important integral part of health care; it has effects on health system performance and clients’ satisfaction [57]. Effective inter-personal communication during childbirth creates trust and confidence (resulting from friendliness, privacy and respect) between women and health workers resulting into a more positive and holistic approach to childbirth. It contributes to right diagnosis and treatment outcomes thus saving lives, time as well as resources. Positive childbirth experience improves perceived quality of care among women thus influencing utilization [58].

Gains from using the eCDSS were undermined by the general lack of or poor laboratory examination (urine and blood tests) and poor quality of counselling during ANC; inadequate care and examination of mother, poor monitoring of mother and newborn (very few Partographs were correctly used- 17%); and lack of or poor basic emergency obstetric and newborn care at the sites. These services were below standard even after eCDSS [54].

Sukums and colleagues (2015) report on implementation experiences of the eCDSS and barriers encountered in the intervention sites, explaining the ultimate effectiveness. The low eCDSS effectiveness could be explained by eCDSS design, provider, organizational, process and technological barriers in the use of the system [33–35].

In order to use the system the health workers had to activate it, this might have acted as a deterrent to consistent usage. Some health workers used the system for data storage rather than for clinical guidance in decision making during face-to-face consultations with clients. Availability of only one computer per health facility might also have missed concurrent services. Moreover, it became particularly problematic when a computer broke-down. This happened in one of our study health facilities leaving health workers to use the traditional paper-based guidelines.

Challenges facing the Tanzanian health system present difficulties in effective implementation of the system, especially in rural settings. Basic infrastructure for health is lacking in many rural health facilities. Electricity and internet access are unreliable, there is shortage and poor retention of skilled health workers, there is unreliable communication and transport and irregular supply of essential drugs and equipment. A significant portion of the health budget is donor funded, which threatens sustainability of the health system in adopting and using HITs such as the eCDSS. Several studies have reported challenges threatening effective implementation of HIT in Tanzania and other similar low-income countries [59–62].

Time is a crucial factor to the success of any medical health information technology. It takes time for providers to learn and use a new technology correctly and effectively. This is even more so in rural contexts where most providers are unexposed to modern computer technology [35]. Properly aligning the eCDSS with the health facility resources is crucial to successful implementation [63]. This may imply process changes (new policies and procedures) involving health facility organization and culture. Organizational readiness for change is very important and this will dictate the difference between success and failure of such a system from one context to another. Training, support and careful monitoring are warranted to correct errors and improve performance [35, 63].

Health workers need to be well trained accompanied by supportive supervision and monitoring for successful implementation. Remuneration of health workers’ use of the eCDSS will motivate utilization. As health workers become conversant and the eCDSS is integrated into the routine system these costs may fall as it may not be necessary to remunerate eCDSS use and trainings and supportive supervisions will be less frequent. As most inputs of eCDSS intervention are fixed, marginal cost of eCDSS would be very small promising economies of scale with increased activity level. Moreover, the current rapid technological advancement promises more durable, versatile and cheap devices with a view of eCDSS cost going down in the future.

The ICERs compare favourably to the Tanzanian per capita GDP, based on current prices (between 998.1 and 1005.6 in 2014) [48, 49] and purchasing power parity (between 2591.2 and 2666.7) [49, 50]. This comparison may however be questionable because the WHO cost-effectiveness thresholds are based on Disability Adjusted Life Years (DALYs) as the outcome measure. This study also compares favourably with other interventions for improving quality of maternal care provision in low and middle income countries [64].

Cost-effectiveness of the eCDSS can be improved through several ways. First, setting up strategies to ensure retention of trained health care workers, training is the largest cost component of eCDSS, therefore retention would reduce the cost of training over-time. The problem of health worker retention facing most rural health care centres may be a challenge to eCDSS implementation especially at the beginning before total integration of the system. Second, payment to compensate health workers’ time is essential to motivate eCDSS utilization especially at the outset because the use of the system may be viewed as a double burden by health workers as they already know what to do. Third, the system could be used in other activities besides MNC, for example record keeping, monitoring and reporting.

The eCDSS (with contextual adaptations) was implemented in Ghana, economic evaluation results show that the system managed to identify complications during pregnancy and to marginally reduce labour complications, the ICER was 1142 USD [65].

### Limitations of the study

Due to the small number of health facilities studied the results may not be easily generalizable and may be specific for the study districts. However, it seems acceptable to judge the study sites as being representative for a common resource poor rural health care setting. The retrospective methodology used in estimating cost may be a limitation. Prospective approach would have been more accurate in capturing resource allocation. Regarding quality measurement, independence of observation assumption may have been violated due to the limited number of health workers in our study sites meaning that observed MNC services were usually done by the same pool of personnel. The voluntary nature of participation in the study could have caused selection bias. However the composite nature of the quality scores generated comprehensive indicators of quality at different levels of care in these facilities. The short usage time of eCDSS is also a limitation for cost-effectiveness analysis.

## Conclusions

Despite initial challenges, the eCDSS was successfully adopted and used for MNC. Although the eCDSS did not show significant effectiveness in improving the overall process quality of MNC, it managed to marginally improve individual process quality variables (history taking and continuity of care during ANC and monitoring mother, inter-personal performance and recording during childbirth). eCDSS cost-effectiveness ratios are affected by assumptions on cost and outcomes but compare well to national GDP per capita and similar interventions. The decision whether or not to adopt and scale-up the system is not clear. These findings call for further studies to uncover the long-term cost-effectiveness of the eCDSS using a larger sample size for the health facilities and observation study and longer timeframe to allow effects to be captured. Realization of gains from eCDSS requires effective implementation and an enabling healthcare system.

## Additional files

Additional file 1: Table S1: Financial cost of eCDSS intervention in USD, 2009-14. This table shows detailed financial cost of eCDSS implementation. (XLSX 32 kb)

Additional file 2: Table S1: Economic cost of eCDSS intervention in USD, 2009-14. This table shows detailed economic cost of eCDSS implementation (XLSX 31 kb)

## Abbreviations

ANC: Antenatal Care
eCDSS: Electronic Clinical Decision Support System
GDP: Gross Domestic Product
ICER: Incremental Cost Effectiveness Ratio
MNC: Maternal and Newborn Care
QUALMAT: Quality of Maternal Care
USD: United States Dollars
WHO: World Health Organization
